# A Model for Sustainable Curriculum Development in Dentistry

**DOI:** 10.1111/eje.13145

**Published:** 2025-07-04

**Authors:** Jonathan Dixon, Nicolas Martin, Sibylle Vital, Julia R. Davies, Denis Murphy, James Field

**Affiliations:** ^1^ University of Sheffield Sheffield UK; ^2^ Université Paris Cite Paris France; ^3^ AP‐HP, Department of Odontology Louis Mourier Hospital Colombes France; ^4^ Malmö University Malmö Sweden; ^5^ Association for Dental Education in Europe (ADEE) Dublin Ireland; ^6^ Cardiff University Cardiff UK

**Keywords:** curriculum, curriculum development, dental, environmental sustainability, oral health professional, sustainable

## Abstract

Oral health professional curricula require continuous evolution to meet the needs of the population. The development of existing curricula to incorporate new topics is rarely considered in the dental education literature, and practical guidance will support educators in achieving changes locally. This paper aimed to present an evidence‐based curriculum development model grounded in current dental and oral health professional education practices. To illustrate and validate this model, the integration of environmental sustainability (ES) into the curriculum will be used as a case study, offering practical insights into a real‐world curriculum development process.

## 1 Introduction

Oral health professional (OHP) curricula must be open, transparent and continuously developed to reflect changes, both in the profession and societal needs. The overarching goal of OHP education is to produce high‐quality healthcare professionals. While programme‐level learning outcomes might not often change, the specific knowledge, skills and behaviours to achieve these outcomes are frequently revised and amended [1, 2]. Examples of recent changes include the addition of inter‐professional education into the curriculum, the use of hybrid teaching modalities and the uptake of digital dentistry. Curriculum development in OHP programmes is typically driven by the following:

- New developments in dentistry and oral healthcare [3, 4, 5, 6, 7];
- Developments in educational rationale and innovation [8, 9, 10, 11];
- Changes in society, stakeholder priorities and global influences [12, 13, 14, 15, 16, 17, 18].

Occasionally, large‐scale curriculum changes are planned after an extensive review of all curricular elements. In this respect, change often involves a complete rebuild of the curriculum and, also perhaps, a change in overall ideology or philosophy for the curricular approach. Regardless, reviewing the entire curriculum is highly resource‐intensive, and regularly repeating this process is unsustainable in most contexts. A more flexible and manageable curriculum development process is necessary to continually update *existing* curricula to ensure they are fit for purpose. Curriculum development in most contexts can be considered a cyclical, planned and progressive process of improving existing educational practices and curricula.

While curriculum development is an integral part of OHP education, there is limited evidence to describe *how* this is, or should be, achieved. Numerous published models describe curriculum development in health professional education [4, 19, 20, 21]. However, most refer to large‐scale curriculum change and compare different curriculum philosophies. Kern's model of curriculum development [21] refers to the development of existing curricula and is frequently cited in health professional education literature. While the philosophy of Kern's model is still relevant, many of the procedural steps and terminology might now be considered outdated. The sheer volume and range of topics held within curricula means that a more efficient and pragmatic approach is required to avoid stagnation.

This paper aimed to present an evidence‐based model for curriculum development grounded in current dental and OHP education practices. To illustrate and validate this model, the integration of environmental sustainability (ES) in the curriculum is used as a case study, offering practical insights into a real‐world curriculum development process.

## 2 A New Model for Curriculum Development in Oral Health Professional Education

**FIGURE 1:**
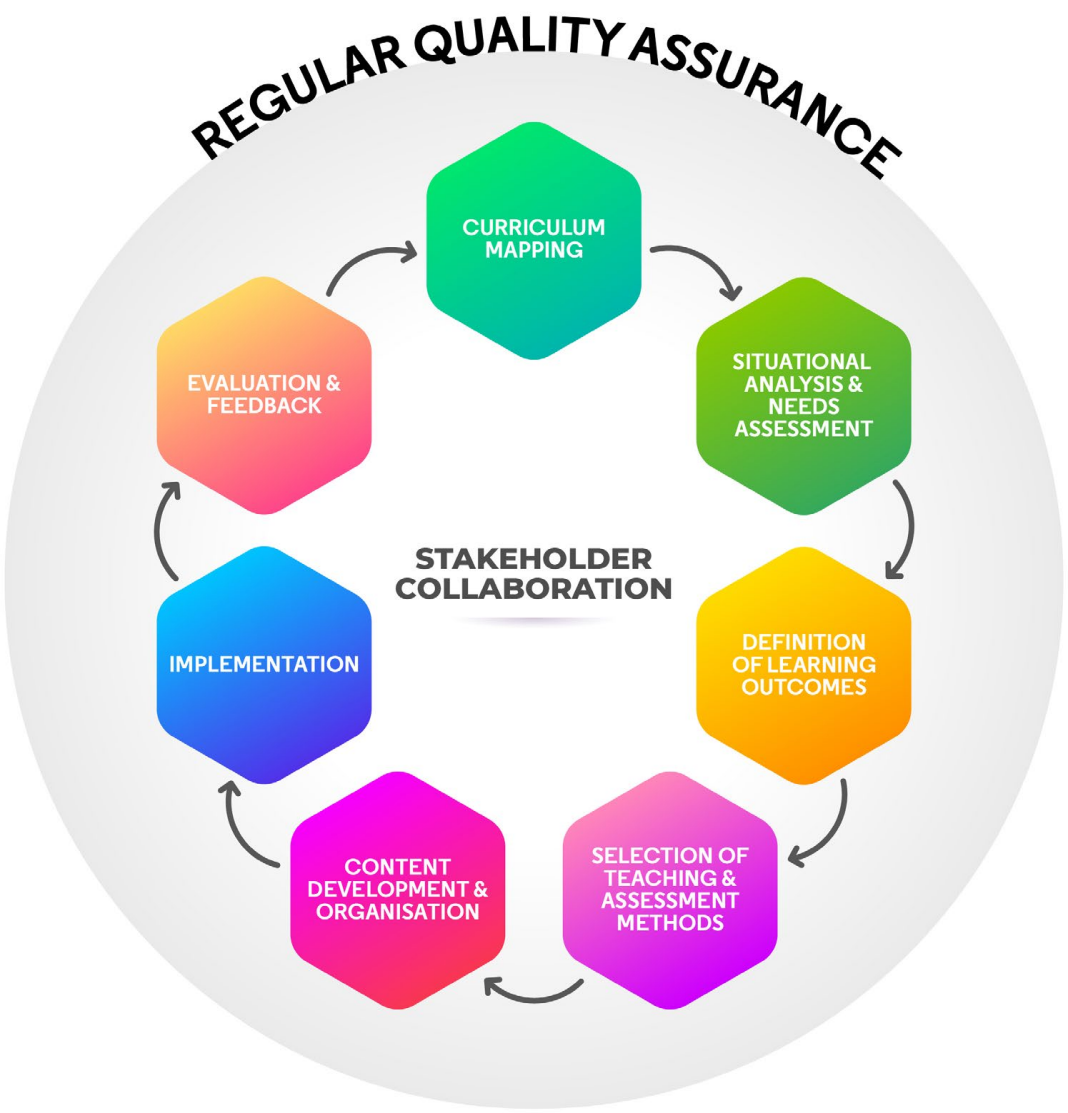
A contemporary model for curriculum development in oral health professional education.

This model for curriculum development builds upon the philosophy and foundations of Kern's model—but also emphasises the critical importance of stakeholder collaboration and regular regulatory, institutional, school and programme‐level quality assurance (Figure 1). The model comprises seven components that operate as part of an iterative cycle:

1. Curriculum mapping
2. Situational analysis and needs assessment
3. Definition of learning outcomes
4. Selection of teaching and assessment methods
5. Content development and organisation
6. Implementation
7. Evaluation and feedback

Curriculum development should not be an isolated activity with a defined endpoint; it is a cyclical and continuous process where evaluation and feedback inform future practice [22]. Stakeholder input and collaboration are critical across all aspects of the process. Additionally, regular quality assurance processes allow for a critical inquiry into the curriculum through multiple lenses. Both stakeholder collaboration and quality assurance are fundamental components of the curriculum development process and, therefore, contribute to all individual stages described below.

### 2.1 Curriculum Mapping

Curriculum mapping is the start and end point of the curriculum development cycle. A completed map allows the school to demonstrate how curriculum elements align with other internal and external frameworks. Curriculum mapping allows for the straightforward interrogation of learning outcomes to identify opportunities for curriculum development. It enables cross‐referencing to avoid duplicating teaching/assessment events and determine whether new changes will make other learning outcomes redundant. This overview also allows educators to identify the institutional thresholds for change and create a realistic and achievable action plan.

**FIGURE 2:**
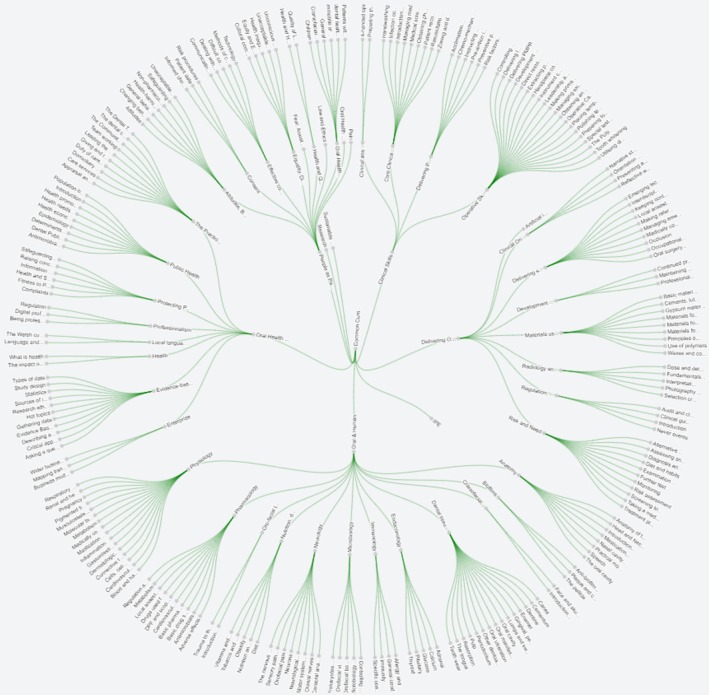
An example of a curriculum map from the School of Dentistry, Cardiff University, which allows students and staff to freely interact with structure, components and mapping details.

**FIGURE 3:**
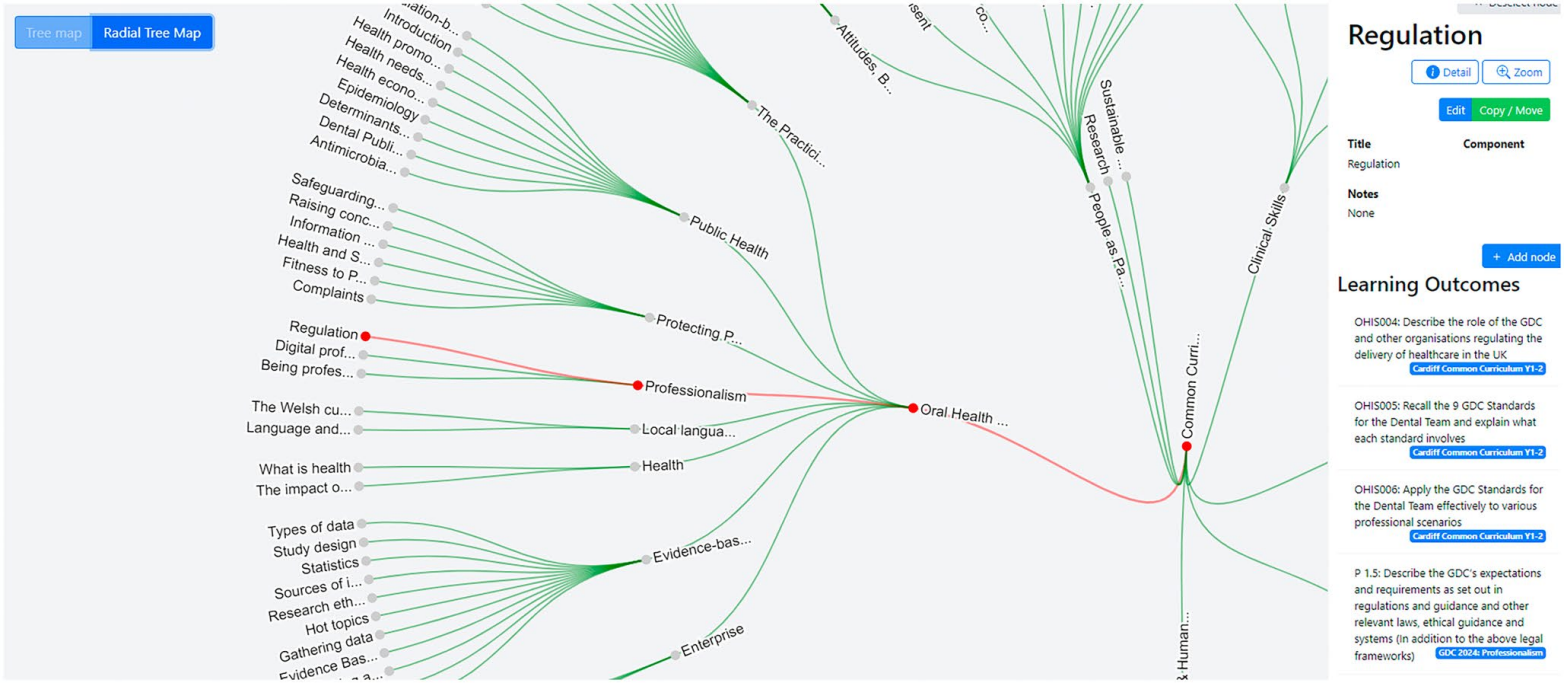
One strand of the curriculum map demonstrating the positioning of the learning outcomes within curriculum themes. Each learning outcome is mapped to the Graduating European Dentist and national regulator frameworks, making it easy to see the value of each outcome, and its position in the curriculum.

Traditionally, curriculum maps have been created in databases or spreadsheets, although this often restricts access to a handful of staff, meaning that the mapping cannot be viewed or interrogated by wider staff or students. More recently, curriculum mapping tools offer better interfaces for building, displaying, mapping and searching learning outcomes. These systems can provide educators and students with rapid access to the curriculum structure and content at any time, allowing direct visualisation of the location of learning outcomes in the curriculum and the methods used to teach and assess these [23]. Additionally, curriculum mapping provides complete transparency and visibility of the current situation for all stakeholders [24]. As these systems develop further, their utility for students also increases. Figures 2 and 3 show examples of curriculum structure at various levels of detail, through the CAFS portfolio system (Invent Partners) at the School of Dentistry, Cardiff University. All stakeholders can freely interact with the map, browse the structure or search for content by keyword(s). All learning outcomes are mapped to the General Dental Council (GDC) and Graduating European Dentist (GED) frameworks, but can also be mapped to any other framework, such as a local graduate attributes framework. The content can be interrogated with any learning outcome (internal or external) as a starting point. Ultimately, students are able to tag their portfolio reflections with relevant learning outcomes, adding even more value to the mapping process.

### 2.2 Situational Analysis and Needs Assessment

A case for change must be established from a comprehensive analysis of the existing situation and the needs of multiple stakeholders. Following recommended quality assurance standards, current practice should be compared to an ideal example, with any differences representing the needs assessment [21, 25]. Early or ongoing consultation with stakeholders and ‘critical friends’ will provide a different context for the planned changes and raise potential barriers to change [26, 27]. Various methodologies may be used to establish a needs assessment, including quantitative (survey questionnaires, clinical performance data) and qualitative (focus groups, interviews) data collection from internal and external data sources, literature searches and policies from regulators [6].

In addition to establishing a case for change, it is necessary to perform a comprehensive analysis of the situation in which change will occur. This should include the following:

- Resource analysis: including staffing numbers and ratios, staff skills and experience, physical space, clinical/simulation unit capabilities, available technologies, infrastructure and funding.
- Performance analysis: considers student and staff performance in the area of interest and compares it with an established standard.
- Content analysis: current curriculum content in the area of interest and potential opportunities for reinforcement. The GED curriculum library is a useful way to identify existing research and scholarship in the area of interest: https://adee.org/graduating‐european‐dentist/graduating‐european‐dentist‐curriculum/ged‐library.
- Training suitability analysis: is the graduating workforce appropriate for existing and future population needs? Liaison with workplace‐based providers and departments of health where appropriate.
- Cost–benefit/utility analysis: selecting cost and time‐effective interventions to maximise benefit.
- Student voice: student perceptions of the current context, understand opportunities and challenges to achieving the desired change.

The results of these processes provide significant insight into the drivers for change and the key challenges to implementing a curriculum development project. Identifying these two competing ‘forces’ from the start of the process is critical to producing a viable pathway for success. Lewin's force field analysis theory is used widely in social science for change management and states that existing ‘driving’ and ‘restraining’ forces are balanced to reflect the current situation [28]. An imbalance is needed to deliver change by accelerating the ‘driving’ forces and limiting the impact of the ‘restraining’ forces. Producing a vision for the curriculum development process and incorporating a strategy to manage these contextual factors are essential starting points for any initiative.

### 2.3 Definition of Learning Outcomes

Defining the overarching educational goals for the intervention provides clarity for all stakeholders and produces a vision that should be compatible with the existing situation and resource availability [26, 27]. Here, the focus is on programme‐level learning outcomes initially, ensuring that the topic in question is respected more widely within the intended graduate outcomes. This also allows the wider education team, the institution, and other external stakeholders to better understand the focus of the change. The team can then focus on discipline‐level or learning event‐level outcomes. In this context, learning outcomes are recognised as the core component of outcome‐based curricula that are widely adopted in health professional education [1, 2, 29, 30]. Learning outcomes have been defined as ‘a series of individual and objective outcomes, with shared ownership between students and staff, designed to facilitate the learning and assessment process’ [31]. They should state the expected learning that the student must achieve and use meaningful verbs appropriate to the learning mode according to Bloom's domains [32].

If the planned curriculum development requires new learning outcomes, they should be clear and concise to provide structure for students and educators. Published curriculum documents may be used, or the dental school can develop learning outcomes internally. When developing new learning outcomes, stakeholder collaboration is essential, and using consensus‐based methodologies such as the Delphi process is recommended [4, 33, 34]. Within this process, it is recommended that educators are given opportunities to provide qualitative feedback on proposed curriculum development, such as the importance of the learning outcome within the curriculum (essential, important or aspirational), and how difficult it is to teach or assess. This will allow curriculum leads to determine not only whether the outcome should be included, but when and where in the wider course of study. It allows assessment leads to better standard‐set the assessments by understanding which elements are essential or more aspirational. Difficult concepts can be broken down or deconstructed to introduce students to them earlier. Finally, educators can also work together collaboratively to overcome topics that are difficult to teach or assess. There are several recent examples of these approaches within the literature [34, 35, 36].

Sustainability and efficiency must be embedded into all processes. To reduce the risk of exponentially increasing learning outcomes, existing learning outcomes may be modified and adapted in many contexts to include the new area of focus. Importantly, curriculum mapping must also ensure that redundant learning outcomes are removed.

### 2.4 Selection of Teaching and Assessment Methods

After the intended learning outcomes for the new curricular elements have been defined, the most suitable teaching and assessment methods should be selected [21]. The methods employed must be appropriate for the defined cognitive level of the learning outcome [32, 37]. Constructive alignment between teaching and assessment methods and defined learning outcomes is a core approach used in contemporary dental and OHP education [38]. This ensures complete alignment in all critical elements of the formal curriculum; the learning outcomes must inform the teaching and assessment methods, and the assessment methods must reflect the learning outcomes and the teaching received. Considering the overloaded nature of OHP curricula, in many cases, it may be preferable to amend existing teaching and assessment methods to incorporate the desired change. However, innovative teaching and assessment methods may need to be developed for more novel subjects.

### 2.5 Content Development and Organisation

Teaching content should be written to align with the learning outcomes and teaching and assessment methods selected in the previous step of the curriculum development cycle. The material should be written jointly, rather than in isolation, so that module, topic or programme leads have a shared understanding of what is delivered and how. This provides an opportunity for building efficiencies across a range of topics and identifies opportunities to teach and assess elements concurrently. Those overseeing the curriculum should also advise on regular content that they expect to see referred to during the teaching, often threshold concepts or overarching themes, such as ES, inter‐professional education or research.

Educators and students must be empowered to take ownership of the change to maximise efficiency and impact [27]. Staff and faculty education events must be in place to provide the relevant knowledge to support the change and allow educators to tailor their content to the changing context. Student buy‐in is equally important, and co‐creating learning content is a valuable way to collaborate and contribute to the goals of the proposed intervention [39]. Alongside the organisation of content, effective and efficient administration around learning outcomes should be managed. This includes the delivery, mapping, storing, accessibility and aligned assessments for each learning outcome. Effective cross‐referencing of new learning outcomes with the existing curriculum framework is essential to avoid duplication.

### 2.6 Implementation

Implementing the proposed curriculum development initiative requires strong leadership and explicit communication of the outcomes from all the previous stages to gain approval and support from school and university committees. Relevant bodies must also approve the administrative aspects of the changes. The new curriculum intervention must then be clearly communicated to staff, students and other stakeholders, with open streams for discussion. There must be an understanding that significant time and resources are required to achieve change, and this will be an iterative process with continuous development [40]. Piloting the intervention may help gain insight into its feasibility and uncover any barriers [21]. Additional consideration needs to be made for teaching out old curricula and roll‐out plans—these need to be carefully considered from the start of implementation.

### 2.7 Evaluation and Feedback

Evaluating the outcome of the pedagogic intervention is a critical element of the curriculum development cycle. An exploratory approach through educational research can reveal the success of the new curriculum intervention. Accessible, representative and anonymous evaluation streams must be available to provide constructive feedback on the process adopted by the curriculum developers. It must be ascertained whether the intervention has been useful, time‐ and cost‐effective, and feedback should inform future developments [41]. Input from all stakeholders of OHP education should be sought where possible. Student feedback plays a significant role in higher education institution metrics as the recipients of education. Equally, constructive feedback from staff, as the deliverers of the curriculum, should lead to refining the change. External quality assurance processes, including school visits, regulator assessments and external examiners, can provide a different perspective on developments in the curriculum. All evaluation processes should feed back into the needs assessment, which emphasises the continuous cyclical nature of curriculum development. Obtaining pre‐intervention data (such as that discussed in Stage 2: Situational Analysis and Needs Assessment) are critical in order to quantitatively or qualitatively demonstrate the effectiveness and impact of the change.

## 3 Case Study: Embedding Environmental Sustainability Into the Oral Health Professional Curriculum

ES is an emerging societal challenge that mandates all sectors to reconsider their practices. Dentistry has been highlighted as an area of concern due to the significant environmental impacts of oral healthcare provision, principally resulting from patient travel, staff commuting, procurement of dental products, energy and water consumption and waste production [42, 43, 44]. Multiple stakeholders, including students, staff and national regulators, have recognised the need to embed ES in undergraduate OHP curricula [2, 12, 45, 46, 47, 48, 49, 50].

Multiple educational research studies have been conducted to validate the proposed curriculum development model by assessing the process of integrating ES into the undergraduate dental and OHP curriculum at the University of Sheffield. The following sections will explore the curriculum development cycle through the lens of ES and highlight these studies as illustrative examples of its practical application.

### 3.1 Curriculum Mapping

Although not a specific research outcome, a curriculum mapping process was performed to identify existing educational practices and opportunities to incorporate new concepts such as ES. Reviewing the existing curriculum in diagrammatic form allowed visualisation of the placement and relationship of different curricular elements and helped shape the strategy developed in future stages. Holding all teaching and assessment events in a single database (Microsoft Access) facilitated rapid review and enabled direct mapping of ES to existing events.

### 3.2 Situational Analysis and Needs Assessment

An extensive situational analysis and needs assessment was achieved through various educational research projects. A scoping review and pan‐European survey were employed to better understand the state of OHP education in Europe [51, 52, 53]. This research provided critical insight into existing curriculum structures and practices across a continent that all follow the same professional qualifications directive [54, 55]. This research and the development of the vision for OHP education provided a stakeholder‐agreed gold standard that the local curriculum should aspire to reach [51, 52, 53, 56]. Specifically for ES, current educational practice, drivers and barriers were explored nationally and across Europe through mixed methods approaches [40, 57]. The results of this research provided an important understanding of the current situation and facilitated local planning and identified key barriers to change.

Locally, the student voice, as a key stakeholder of OHP education, was included through a survey of student opinion regarding the importance and relevance of ES in the undergraduate curriculum [47]. This augmented previous research findings that demonstrated significant support for including ES in the curriculum from multiple stakeholders, including educators, students, educational organisations and national regulators [2, 12, 35, 45, 46, 48, 58, 59]. From this point, the need to embed ES in the local curriculum was clearly defined.

### 3.3 Definition of Learning Outcomes

The learning outcomes for this curriculum initiative were selected from the recent consensus paper published by the ADEE ‘Sustainability in Dentistry’ special‐interest group [35]. A pan‐European consultation was undertaken to write specific learning outcomes for ES, and the group developed seven new learning outcomes. Additionally, seven learning outcomes from the original GED framework were amended to include ES [1]. These outcomes have now been incorporated into the online version of GED (https://adee.org/graduating‐european‐dentist/graduating‐european‐dentist‐curriculum) and are presented in green font [60]. The GDC in the United Kingdom included two of these learning outcomes in the recent curriculum update, the Safe Practitioner framework [2]. As the GDC is the national regulator of the United Kingdom, these two learning outcomes for ES needed to be incorporated into the plan, and one additional learning outcome from the ADEE work was included. The learning outcomes selected were the following:

- Describe the main principles relating to sustainable oral health care, both environmentally and in terms of patient compliance and the factors that might affect implementing a sustainable approach.
- Evaluate and apply the evidence base in relation to the environmental impacts of common treatment methods and approaches to the delivery of oral healthcare.
- Develop effective patient‐specific strategies for preventive oral health, reducing the need for recall, operative intervention and material use.

### 3.4 Selection of Teaching and Assessment Methods

The teaching and assessment methods proposed for ES were developed from the learning outcomes to ensure constructive alignment [38]. Two sources informed the final methods selected. Firstly, The ADEE ‘Sustainability in Dentistry’ special‐interest group proposed teaching and assessment methods for each learning outcome [35]. Secondly, the FDI (World Dental Federation) produced evidence‐based learning content for ES in the form of a Massive Open Online Course (MOOC), which aims to educate the profession at all levels [61].

To explore stakeholder opinions locally, focus groups were conducted with educators and students to identify opportunities to teach and assess each learning outcome within the local curriculum [62]. This research provided excellent insight into the possibility of *modifying* the existing teaching and assessment methods without adding multiple new events. This approach is considered beneficial due to the reported challenges with the overloaded curriculum [45, 57]. Additionally, the need to teach ES across all disciplines with practical application was emphasised. Considering the sources described and the outcome of the focus group research, four teaching methods were planned across all years of the undergraduate dental and OHP curriculum:

- Massive Open Online Course (MOOC) FDI—Sustainability in Dentistry: an online course with an approximate learning time of three hours [61]. Embedded into 1st‐year dentistry and 2nd‐year dental hygiene and therapy programmes.
- Standalone lecture titled ‘Environmental Sustainability in Dentistry’: a one‐hour lecture providing baseline knowledge regarding ES and key mitigation strategies. Embedded into 2nd‐year dentistry and 1st‐year dental hygiene and therapy programmes.
- Embedding ES content into existing learning and teaching events across all disciplines: through inclusion ‘content statements’ as a single or group of slides [62]. Embedded into existing theoretical and practical teaching events from year 1 to 4 of the dentistry programme.
- Clinical case‐based discussion including ES: clinical scenarios that incorporate elements of ES alongside high‐quality patient care. A single two‐hour session that included multiple cases in the field of restorative dentistry, endodontics, prosthodontics, periodontology and cariology. Embedded into the 4th‐ and 5th‐years of the dentistry programme.

Existing methods of assessments were augmented to include ES components, including OSCEs, written examinations and online quizzes. As this curriculum development project progresses and students receive more teaching relating to ES across all years of study, ES will be incorporated to a greater extent into other assessments.

### 3.5 Content Development and Organisation

Given the methods selected and educators' reported unfamiliarity with the topic of ES, it became increasingly clear that evidence‐based and subject‐specific content needed to be developed in the field of ESD [40, 45, 48, 57, 58]. A research methodology grounded in exploring the evidence base and achieving stakeholder consensus resulted in the development of 44 content statements for ESD [62]. The content statements were mapped to all curriculum subjects and validated through subject‐expert consultation. This work enabled educators of all disciplines to identify and use evidence‐based content on ES that was relevant to their area of expertise. Slide decks were developed for all disciplines and shared with educators. A collaborative approach between curriculum managers and educators was completed to identify existing teaching events that would benefit from the inclusion of ES, and the educators received the evidence‐based content that could be added to their teaching in the form of one or multiple slides. This work has now been adopted by the FDI World Dental Federation and the slides decks are available as open‐access resources (https://www.fdiworlddental.org/education‐resources‐sustainable‐dentistry).

### 3.6 Implementation

The work completed in the previous steps informed a plan to embed ES in the local curriculum. The strategy was agreed locally through the relevant quality assurance processes. The curriculum development project commenced in September 2024, and the planned teaching events were delivered to all year groups. Effective organisation and clear communication of the planned changes was critical. Additionally, it was important to provide support and reassurance to educators and students during this change.

### 3.7 Evaluation and Feedback

Multiple methods of evaluation and feedback were used to review the process and outcome of embedding ES in the curriculum. To measure the impact of the teaching interventions on student awareness, attitudes and knowledge of environmental sustainability in dentistry (ESD), all students were invited to complete baseline and post‐intervention surveys [63]. This research demonstrated that longitudinally embedding ES across all years of the undergraduate dental programme resulted in significant positive changes in OHP students' awareness of ESD, attitudes towards ESD, general pro‐environmental attitudes and knowledge of ESD. The views of educators and curriculum managers with respect to the change were established through anonymous surveys.

A student‐led audit tool, the Planetary Health Report Card (https://phreportcard.org/dentistry/), was implemented to enable the student cohort to grade the school's performance concerning environmental sustainability across numerous domains, including the curriculum. Finally, student satisfaction surveys were completed as part of routine internal quality assurance to review student perceptions of this curriculum development initiative. These findings provided critical insight into the intervention's success and helped improve this process for the next cycle.

## 4 Conclusions and Recommendations

A perpetual curriculum development cycle is critical to ensure OHP education remains fit for purpose and is able to meet the needs of a frequently evolving profession. This paper proposes a new model of curriculum development that is better suited to current OHP educational practices. This approach utilises contemporary educational terminology and better reflects the importance of stakeholder collaboration and quality assurance processes. It highlights the cyclical and iterative nature of curriculum development.

The following recommendations are made for the implementation of this model:

- Deploy an easy‐to‐use and effective curriculum mapping software to give critical insight into the content of existing curricula including how they map to internal attributes, external curricula or regulatory requirements.
- Conduct extensive local and regional needs assessments to identify the drivers for curriculum development and establish a gold standard of practice.
- Review the constraints of the existing curriculum through a situational analysis to identify and predict challenges to the proposed development initiative.
- Carefully and objectively select learning outcomes for the planned change and ensure sustainability by modifying existing learning outcomes and removing redundant items.
- Constructively align selected learning outcomes with teaching and assessment methods and explore opportunities to augment existing events.
- Employ multiple methods of evaluation to review the impact of the intervention through the lens of all relevant stakeholders.

## Conflicts of Interest

The authors declare no conflicts of interest.

## Data Availability

Data sharing not applicable to this article as no datasets were generated or analysed during the current study.
